# Population pharmacokinetics of rivaroxaban after transjugular intrahepatic portosystemic shunt

**DOI:** 10.1007/s00228-026-04034-6

**Published:** 2026-03-24

**Authors:** Min Jia, Yiming Chai, Yuan Gao, Changyou Jing, Kunlei Zhu, Tong Zhu, Liang Wang, Aili Sun, Jun Yang, Yanqiu Zhu, Yingmei Feng, Yu Cao, Jianjun Li

**Affiliations:** 1 Beijing Institute of Hepatology, Beijing Youan Hospital, Capital Medical University, No. 8, Xi Tou Tiao, Youanmen Wai, Fengtai District, Beijing, 100069 China; 2 Quality Management Office for Clinical Research, Beijing Youan Hospital, Capital Medical University, No. 8, Xi Tou Tiao, Youanmen Wai, Fengtai District, Beijing, 100069 China; 3 Zhongchuang Huiming (Beijing) Medical Technology Co., Ltd, No. 223-224, 5th Floor, No. 181, Laogongying Village, Liulimiao Town, Huairou District, Beijing, 101405 China; 4 Liver Disease Center, Beijing Youan Hospital, Capital Medical University, No. 8, Xi Tou Tiao, Youanmen Wai, Fengtai District, Beijing, 100069 China; 5 Hepatic Disease and Oncology Minimally Invasive Interventional Center, Beijing Youan Hospital, Capital Medical University, No. 8, Xi Tou Tiao, Youanmen Wai, Fengtai District, Beijing, 100069 China; 6 National Clinical Trial Institution for Drugs, Beijing Youan Hospital, Capital Medical University, No. 8, Xi Tou Tiao, Youanmen Wai, Fengtai District, Beijing, 100069 China; 7 Department of Science and Technology, Beijing Youan Hospital, Capital Medical University, No. 8, Xi Tou Tiao, Youanmen Wai, Fengtai District, Beijing, 100069 China; 8 Department of Clinical Epidemiology, Beijing Youan Hospital, Capital Medical University, No. 8, Xi Tou Tiao, Youanmen Wai, Fengtai District, Beijing, 100069 China

**Keywords:** Rivaroxaban, Population pharmacokinetics (PopPK), Transjugular intrahepatic portosystemic shunt (TIPS), Model-informed dosing

## Abstract

**Purpose:**

Rivaroxaban, a new direct oral anticoagulant (DOAC), has demonstrated better efficacy and safety than traditional anticoagulants. Individualized anticoagulation is crucial in patients with a transjugular intrahepatic portosystemic shunt (TIPS) and portal vein hypercoagulability to avoid stent thrombosis. As TIPS shunts portal blood directly into the systemic circulation and may substantially affect the first-pass extraction/distribution of direct oral anticoagulants, no population prospective pharmacokinetic studies have been published in patients undergoing TIPS placement so far. The goal of this study was to establish a PopPK model for rivaroxaban in post-TIPS patients, investigate clinical covariate effects on PK, and provide an optimized dosing regimen.

**Methods:**

In this single-centre prospective study, 39 adult patients underwent TIPS and received rivaroxaban 5 or 10 mg once daily thereafter. Population PK analysis was conducted in NONMEM with 131 plasma concentrations available from 38 evaluable patients (median age: 56 years; median body weight: 63.8 kg). Parameterized model was then applied to simulate steady-state exposure of 5, 7.5, 10, and 15 mg once daily regimen.

**Results:**

The final PopPK model was based on a one-compartment with sequential zero-order followed by first-order absorption kinetics, and included the effects of an absorption lag time and linear elimination. The apparent clearance and volume of distribution were 7.48 L/h and 4.75 L, respectively. Based on patient-specific simulations of 38 subjects, at the 5 mg/day dose, an exposure within predefined limits was potentially preserved in 96.7% of the cases vs. 62.5% for the 10 mg/day (thresholds: AUC_ss,24_ ≤ 1.77 mg·h/L and C_max, ss_ ≤ 140 µg/L). This trend was confirmed by Monte-Carlo simulations in 1,000 virtual patients and suggests TDM when the higher dosage is given.

**Conclusions:**

This PopPK model provides an initial characterisation of rivaroxaban disposition in post-TIPS patients and reveals a markedly reduced apparent V/F relative to non-TIPS populations. The 5 mg once-daily regimen is generally safe, while the 10 mg dose may be considered with therapeutic drug monitoring to account for substantial inter-individual pharmacokinetic variability in post-TIPS patients.

**Trial registration:**

Chinese Clinical Trial Registry, ChiCTR (ChiCTR2300073784); registered 20 July 2023.

## Introduction

Rivaroxaban, a direct oral anticoagulant (DOAC), has gradually replaced conventional anticoagulation with warfarin due to its favorable safety and efficacy profile in the prophylaxis and treatment of venous thromboembolism (VTE) [1–11]. In particular, rivaroxaban has potential benefits of oral dosing, which offers convenience compared with subcutaneous administration required for heparins; such a wide therapeutic range that the need for routine international normalized ratio measurements can be avoided, as is needed for warfarin treatment; and inconspicuous drug-drug interactions, which would decrease combined anticoagulation therapy risks in patients receiving more than one medication, potentially increasing patient acceptability [3, 5, 12]. Metabolism and excretion of rivaroxaban are primarily through two pathways: hepatic metabolism with CYP 450 enzymes (predominantly CYP3A4 and CYP2J2) to biologically inactive metabolites, and renal excretion as unchanged drug via the kidneys where active tubular secretion is mediated by transport proteins including p-glycoprotein (ABCB1 gene) and breast cancer resistance protein (ABCG2 gene) [13].

Transjugular intrahepatic portosystemic shunt (TIPS) is a useful treatment for complications of portal hypertension, particularly acute variceal bleeding and refractory ascites. However, in the post-TIPS setting many patients develop hypercoagulability because of an underlying condition (e.g., Budd-Chiari syndrome, asplenia) or history of portal vein thrombosis. This hypercoagulability dramatically increases the risk of stent thrombosis or failure, and thus prophylactic anticoagulation is required in such high-risk patients [14, 15]. However, the TIPS effect of change in hepatic blood flow might greatly affect the pharmacokinetics of drugs and thus their effective and safety [16]. The literature also shows that TIPS could change the bioavailability and C_max_ of drugs such as midazolam and caspofungin [16, 17]. Although rivaroxaban is a potential anticoagulant treatment option for this patient population, comprehensive pharmacokinetic information in post-TIPS patients remains incompletely characterized. The previous data on the pharmacokinetics of rivaroxaban in hepatic insufficiency is limited to Child-Turcotte-Pugh class A or B, without taking into account portosystemic shunting; thus it cannot be applied to the patients with TIPS [1]. The present study attempts to fill this knowledge gap by utilizing population pharmacokinetic (PopPK) modeling to describe the inter- and intra-individual variability in rivaroxaban exposure, and systematically investigate the influence of specific clinical characteristics such liver functions and renal function on rivaroxaban pharmacokinetics. We hope that such modeling framework could be used to individualize rivaroxaban dosing and improve clinical decisions related to anticoagulation therapy post TIPS procedure.

## Methods

### Study design, population and treatment

This prospective, single-center study was conducted in the Hepatic Disease and Oncology Minimally Invasive Interventional Center ward of Beijing Youan Hospital, Capital Medical University, Beijing, China between July 2023 and March 2025. Adults aged 18 − 70 years who had undergone TIPS and were scheduled to receive postoperative rivaroxaban were eligible. Exclusion criteria included: active bleeding; liver cancer or other systemic malignancies; inability to abstain from smoking or alcohol during the study, or any condition deemed by the investigators to preclude participation.

All TIPS operations were performed by experienced interventional radiologists using conscious sedation and local anaesthetic. A transjugular approach was employed under fluoroscopic guidance; the portal vein was punctured, balloon-dilated and an 8 mm PTFE-covered Viatorr stent deployed, to achieve two pre-specified hemodynamic target endpoints: a ≥ 50% decrease in porto-portacaval pressure gradient from baseline and an absolute gradient ≤ 12mmHg.

5 or 10 mg rivaroxaban (Suzhou Third Pharmaceutical Factory Co., Ltd., China) was given orally once a day to TIPS patients at the discretion of the doctors on postoperative day 3. The study pharmacist administered the first observed dose and recorded the exact time of its administration on the case report form.

The study was approved by the Ethics Committee of Beijing Youan Hospital, Capital Medical University. All participants provided written informed consent.

### Blood sampling

All subjects had 3 mL venous blood samples taken at 2, 4 and 24 h (± 0.5 h) following the first identifiable dose. After protocol revision, the last 23 participants had an additional 8 h (± 0.5 h) sample obtained. After centrifugation within 2 h of the blood drawing (3,000 rpm, 10 min, 4 ℃), plasma was aliquoted and kept at − 80 ℃ for analysis.

### Data collection

Demographic and clinical variables—sex, age, height, weight, estimated glomerular filtration rate (eGFR), Cockcroft-Gault creatinine clearance (CrCl), alanine aminotransferase (ALT), aspartate aminotransferase (AST), gamma-glutamyl transpeptidase (GGT), total bile acids(TBA), total bilirubin (TBIL), direct bilirubin (DBIL), alkaline phosphatase (ALP), total protein(TP), albumin (ALB), globulin (GLB), prothrombin time (PT), prothrombin activity (PTA), prothrombin time ratio (PTR), International Normalized Ratio(INR), activated partial thromboplastin time (APTT), activated partial thromboplastin time ratio (APTTR), fibrinogen content (FIB), thrombin time (TT), D-dimer (DD), fibrin degradation products (FDP), antithrombin activity (ATA), Child-Turcotte-Pugh (CTP) classification, and ascites status were extracted from the electronic medical record. The CrCl (mL/min) was calculated using the Cockcroft-Gault equation:

$$CrCl=\frac{\left(140-Age\right)\bullet Weight}{72\bullet{\displaystyle\frac{Scr}{88.4}}}\;\left(if\;male\right)$$

      

$$CrCl=0.85\cdot\frac{\left(140-Age\right)\bullet Weight}{72\bullet{\displaystyle\frac{SCr}{88.4}}}\;\left(if\;female\right)$$

 

Where SCr represents serum creatinine (µmol/L), weight is actual body weight, and age is in years. Descriptive statistics were generated with R v4.3.1.

### Laboratory analysis

Plasma concentrations of rivaroxaban were measured in 100 µL aliquots using a validated LC-MS/MS assay with verapamil as the internal standard. The calibration range was 1 to 1000 µg/L; accuracy and precision (intra-day and inter-day) were < 15%.

### Population PK modeling

NONMEM^®^ software version 7.5.0 (ICON Development Solutions, Ellicott City, MD, USA) with gFortran compiler version 4.6.3 (http://gcc.gnu.org/fortran) and PsN^®^ version 5.2.6 (https://uupharmacometrics.github.io/PsN) were used for non-linear mixed-effects modelling. The first-order conditional estimation method with interaction (FOCE-I), accounting for both residual variability and inter-individual variability interactions, was used to estimate model parameters. According to previous literature on rivaroxaban pharmacokinetics with one- or two-compartment models [18–20], we first compared our dataset to both structural model configurations. Final model choice was based on an overall evaluation of (i) diagnostic plots, (ii) statistical criteria [likelihood ratio test, Akaike information criterion (AIC), Bayesian information criterion (BIC)], and (iii) precision of parameter estimation (evaluated with relative standard error). Besides, the selection of models took into account research aims, model application target, pharmacological characteristics of the drugs.

### Covariate model

Covariates were first included in a forward step-wise manner (*p* < 0.01, ∆OFV > 6.63) and subsequently could be eliminated in a backward manner (*p* < 0.001, ∆OFV > 10.83). The investigated covariates comprised sex, age, height, weight (actual body weight), eGFR, CrCl, ALT, AST, GGT, TBA, TBIL, DBIL, ALP, TP, ALB, GLB, PT, PTA, PTR, INR, APTT, APTTR, FIB, TT, DD, FDP, ATA, CTP classification and ascites status. According to available publications and the rivaroxaban prescribing information, which indicate no food effect on pharmacokinetics, food was excluded from covariate analysis [13, 21]. No co-administered medications acting on P450 enzymes or P-gp inducing/inhibiting drugs were identified among the enrolled patients.

Prescreening of covariates was performed before the final model was built. For continuous covariates, either linear or power function relationships were tested to evaluate their functional link with pharmacokinetic parameters (e.g., clearance, volume of distribution), along with statistical significance based on a general modeling equation:

$$P_i\;=P_{POP}\bullet{(cov_i/cov_m)}^{\theta_{cov}}$$

Where *P*_*i*_ is the individual model-predicted pharmacokinetic parameter (e.g., clearance) for an individual with covariate value *cov*_*i*_, *P*_*pop*_ represents the population central tendency for the pharmacokinetic parameter, individually, *cov*_*m*_ represents the population median value of the covariate and *θ*_*cov*_ represents the covariate effect. Importantly, continuous covariates were median-centered before model inclusion to enhance model stability and the reliability of parameter estimation.

For categorical covariates (e.g., sex, CTP classification), analysis of variance was used to test their effects on target pharmacokinetic parameter estimates. The investigation method is shown in the equation:

$$P_i=\;P_{pop}\left(if\;male\right)$$

$$P_i=\;P_{pop}\cdot\theta\left(if\;female\right)$$

Where *P*_*i*_ represents the population parameter for male or female, *P*_*pop*_ is the typical value of the population parameter, and *θ* is the influence coefficient of the covariate on the parameter.

### Model evaluation

The final model was evaluated using goodness-of-fit (GOF) plots, including four core scatter plots: conditional weighted residuals vs. time, conditional weighted residuals vs. population predicted concentrations, observed concentrations vs. predicted concentrations, and observed concentrations vs. individual predicted concentrations.

To assess model performance, a prediction-corrected visual predictive check (pcVPC) was conducted. This involved generating 1,000 simulated datasets using Monte Carlo simulation and comparing the 5th, 50th (median), and 95th percentiles between observed and simulated data. For adequate model performance, the 5th, 50th, and 95th percentiles of observed concentrations against those from the simulations. A good model fit was indicated when the observed percentiles aligned within the 90% confidence intervals of the corresponding simulated percentiles.

Model precision was further evaluated using a bootstrap method with 1,000 replicates. Each bootstrap sample was reanalyzed in NONMEM, and the resulting parameter estimates were used to calculate medians and 95% non-parametric percentile intervals (PI). These were then compared to the final parameter estimates for consistency.

### Model simulation

We used the final population pharmacokinetic (PopPK) model to simulate rivaroxaban exposure across commonly used dosing regimens. Monte Carlo simulations were carried out on 1,000 virtual post-TIPS patients per dose level (5, 7.5, 10, and 15 mg once daily), with each simulation extended to steady state.

Key parameters assessed included the area under the concentration-time curve over 24 h at steady state (AUC_ss,24_) and the peak concentration at steady state (C_max, ss_). For safety evaluation, simulated exposure distributions were compared against exposure-bleeding relationships reported in the US FDA’s clinical pharmacology review of rivaroxaban, which was based on non-TIPS populations and used here as a surrogate reference [22]. That analysis showed a clear link between drug concentration and bleeding risk. For this study, safety thresholds (AUC_ss,24_ ≤ 1.77 mg·h/L; C_max, ss_ ≤ 140 µg/L) were set according to the approved 10 mg once-daily regimen, extracted using WebPlotDigitizer v4.5 from Supplementary Fig. S1 and were intended to characterize bleeding risk rather than antithrombotic efficacy. Any virtual patient whose predicted exposure exceeded either of these thresholds was flagged as having an unacceptable risk of bleeding.

## Results

### Patient characteristics

We included a series of 39 TIPS patients (64.1% males) with 136 samples. Three samples were invalid (due to protocol deviations or concentrations below the lower limit of quantification), and 133 valid observations from 38 patients remained. Patients had an average ± standard deviation (SD) age of 56.46 ± 10.62 years and a body weight of 63.8 ± 13.1 kg. Ten mg of rivaroxaban was administered once a day to 8 patients and 5 mg/day to 30 patients. The baseline demographic and clinical characteristics of the patients are shown in Table 1.

**Table 1:** Demographic characteristics

Characteristic*	Participants/Patients(*N* = 38)
Age (years)	57(32 − 76)
Weight (kg)	62(47.5 − 100)
Height (cm)	168(155 − 190)
Sex, n (%)	
Female	14(36.8%)
Male	24(63.2%)
Rivaroxaban dose, n (%)	
5 mg	30(79.0%)
10 mg	8(21.0%)
Laboratory measurements	
eGFR (mL/min/L)	108.8(62.9 − 148.5)
CrCl (mL/min)	127.1(50.5 − 341.0)
ALT (U/L)	36(7 − 289)
AST (U/L)	35(9 − 230)
GGT (U/L)	34(11 − 144)
TBA (µmol/L)	61.2(17.5 − 237.9)
TBIL (µmol/L)	25.1(10.6 − 65)
DBIL (µmol/L)	11.9(5.7 − 45.9)
ALP (U/L)	88(31 − 291)
TP (g/L)	56.9(37.6 − 76.8)
ALB (g/L)	29.7(25.2 − 38.7)
GLB (g/L)	26.1(10.7 − 45.7)
PT (s)	11.2(8.9 − 15.4)
PTA (%)	65.4(41.6 − 89.6)
PTR (-)	1.23(0.98 − 1.69)
INR(–)	1.2(0.98 − 1.59)
APTT (s)	43.1(30.7 − 71.1)
APTTR	1.35(0.96 − 2.22)
FIB (g/L)	2.1(1.02 − 3.99)
TT (s)	20.3(17.1 − 23.7)
DD (µg/L)	3.1(0.3 − 8.9)
FDP (mg/L)	13.4(1.07 − 39.2)
ATA (%)	52.2(29.1 − 95.4)
CTP score, n (%)	
A	15 (39.5%)
B	23 (60.5%)
Ascites, n (%)	
None	11 (28.9%)
Mild	16 (42.1%)
Moderate to severe	11 (28.9%)

*ALB* Albumin, *ALP* Alkaline phosphatase, *ALT* Alanine aminotransferase, *APTT* Activated partial thromboplastin time, *APTTR* Activated partial thromboplastin time ratio, *AST* Aspartate aminotransferase, *ATA* Antithrombin activity, *CrCl* Cockcroft-Gault creatinine clearance, *CTP* Child–Turcotte–Pugh, *DBIL* Direct bilirubin, *DD* D-dimer, *eGFR* Estimated glomerular filtration rate, *FDP* Fibrin degradation products, *FIB* Fibrinogen content, *GGT* Gamma-glutamyl transpeptidase, *GLB* Globulin, *TBIL* Total bilirubin, *INR* International Normalized Ratio, *PT *Prothrombin time, *PTA* Prothrombin activity, *PTR* Prothrombin time ratio, *TBA* Total bile acids, *TP* Total protein and *TT* Thrombin time

*continuous variables were presented by median (min − max); Categorical variables were presented by N (%)

### Population PK model

After conducting sensitivity analysis, two influential data points were excluded, resulting in 131 samples from 38 patients used for model development. The data were best described by a one-compartment model featuring sequential zero-order followed by first-order absorption, an absorption lag time, and first-order elimination. Typical parameter estimates (relative standard errors, RSE %) were: apparent clearance (CL/F) = 7.48 L/h (RSE 9.5%), apparent central volume of distribution (V_d_/F) = 4.75 L (RSE 37.4%), absorption rate constant (Ka) = 0.140 /h (RSE 5.97%), duration of zero-order absorption (D1) = 0.831 h (RSE 29.3%), and absorption lag time (ALAG1) = 1.23 h (RSE 20.0%). Inter-individual variability (IIV), expressed as the coefficient of variation, was 61.5% (RSE 24.6%) for CL/F and 83.4% (RSE 51.4%) for V_d_/F (see Table 2).

**Table 2:** Final parameter estimates and bootstrap precision (1 000 samples)

Parameter	Final Estimate	Relative standard error (%)	Bootstrap of the final model		
			median	95% CI (covariance)	95% PI (bootstrap)
Ka (1/h)	0.140	5.97	0.143	0.118 − 0.164	0.127 − 0.175
CL/F (L/h)	7.48	9.52	7.22	5.69 − 8.87	5.85 − 8.99
V_d_/F (L)	4.75	37.4	4.93	0.658 − 9.22	1.54 − 10.3
D1 (h)	0.831	29.3	0.820	0.334 − 1.34	0.401 − 1.44
ALAG1 (h)	1.23	20.0	1.22	0.771 − 1.68	0.622 − 1.58
IIV_CL/F (%)	61.5	24.6	39.0	19.7 − 59.6	22.0 − 62.2
IIV_V_d_/F (%)	83.4	51.4	61.5	-26.3 − 153.4	15.0 − 176.9
IIV_D1 (%)	65.8	69.4	47.6	-38.9 − 128.2	6.13 − 164.8
IIV_ALAG1 (%)	20.4	96.2	4.09	-16.4 − 24.9	0.547 − 29.2
Proportional residual error (-)	0.0825	24.2	0.0650	0.0356 − 0.105	0.0341 − 0.101
Additional residual error (µg/L)	4.08	52.0	4.46	0.0915 − 9.02	0.106 − 8.58

*ALAG1* Absorption lag time, *CL/F* Apparent total clearance, *D1* Duration of zero-order absorption, IIV_ALAG1 IIV on ALAG1, IIV_CL/F IIV on CL/F, IIV_D1 IIV on D1, IIV_V_d_/F IIV on V_d_/F, Ka absorption rate constant, V_d_/F Apparent volume of central compartment distribution

### Covariate analysis

A nominal associated effect of ALT on CL/F (*P* < 0.01) was detected in the univariate screen, and the variance component dropped to 0.0091. However, further forward inclusion did not reach the predetermined significance level (ΔOFV < 6.63; *p* < 0.01). No other demographic, renal, or hepatic variables significantly decreased IIV on any structural parameter; hence no covariate was included in the final model.

### Model evaluation

**Fig. 1:**
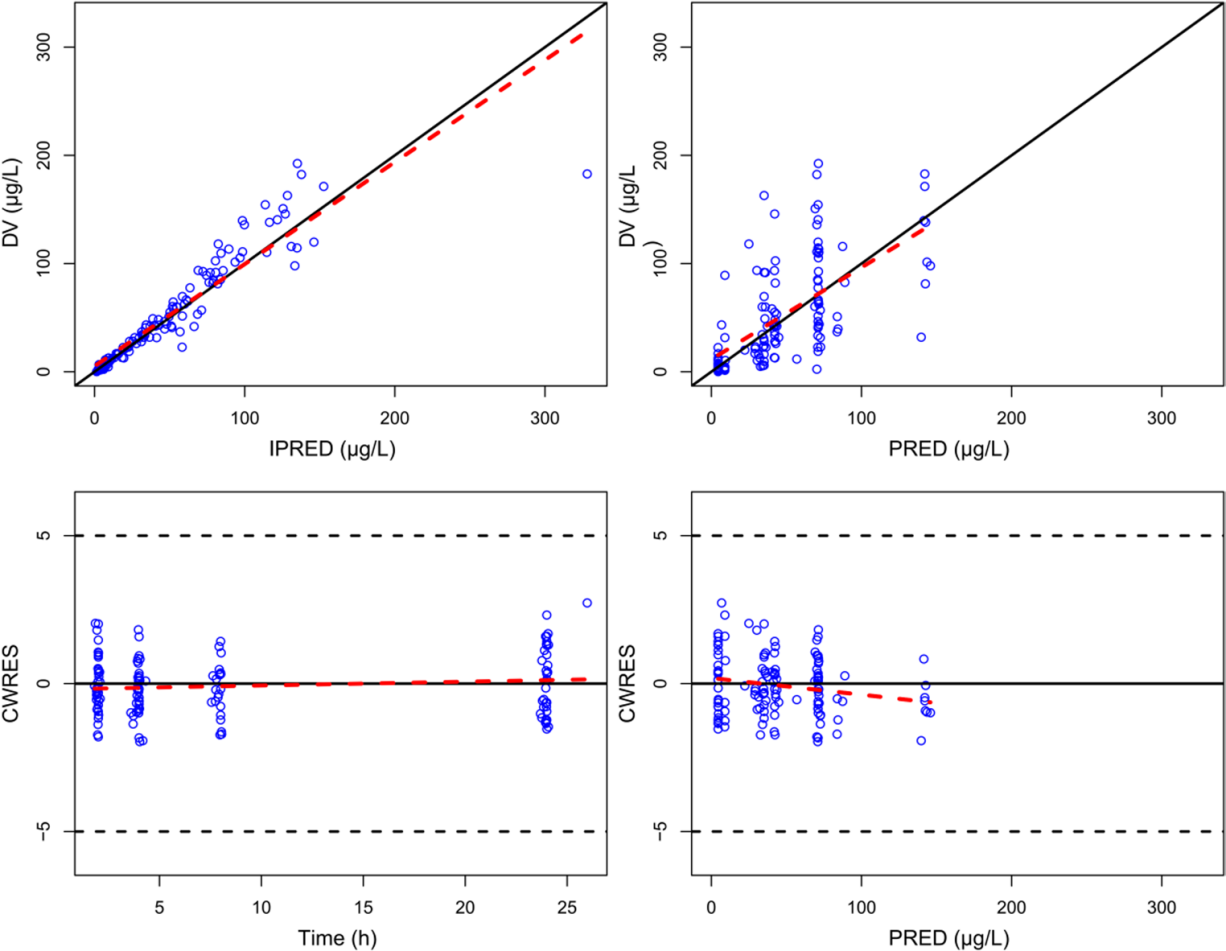
GOF plots of the final model. Top left panel: Individual predicted values versus observed concentrations of rivaroxaban. Top right panel: Population predicted values versus observed concentrations of rivaroxaban. Bottom left panel: Conditional weighted residuals versus time. Bottom right panel: Conditional weighted residuals versus population predicted concentrations. Blue circles indicate observed data points. Black solid lines are identity lines. Red dashed lines are trend lines. Black dashed lines indicate the threshold of ± 5

**Fig. 2:**
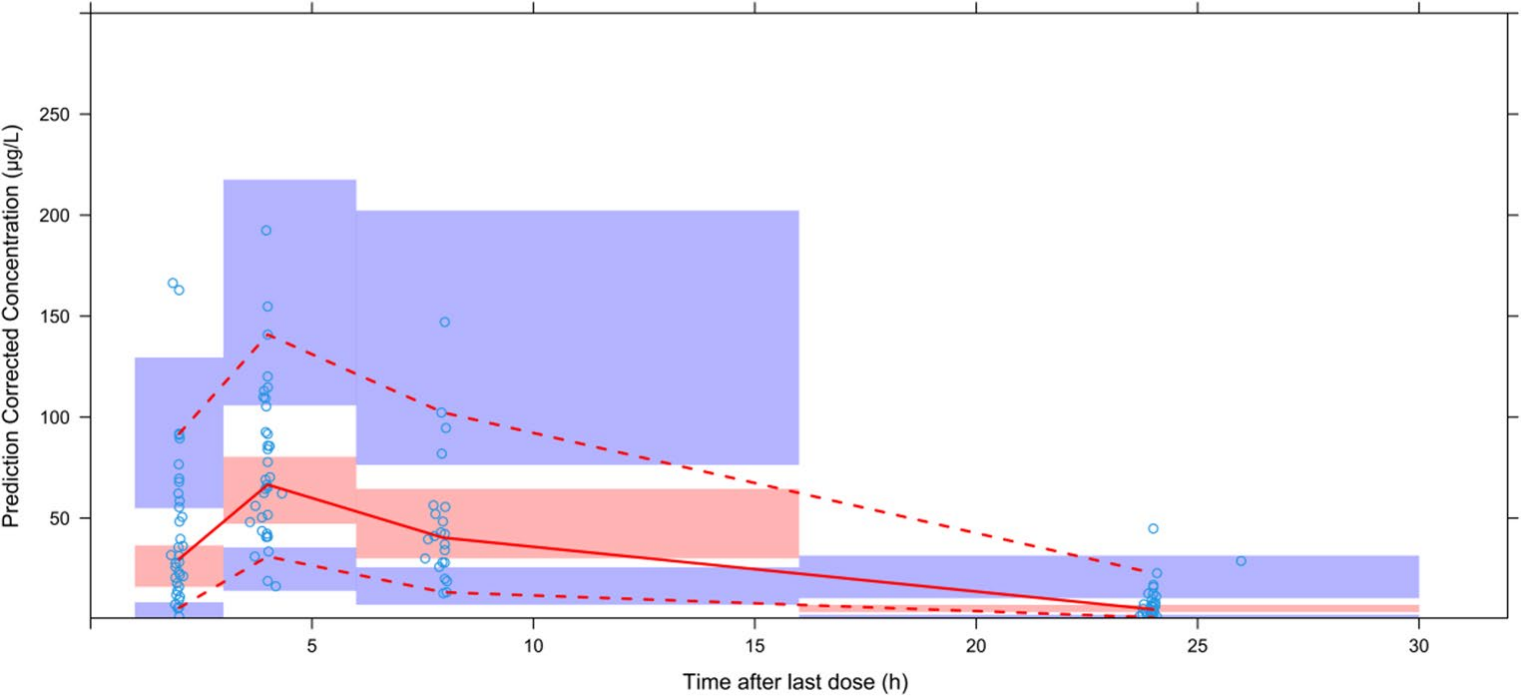
pcVPC plot of the population pharmacokinetic model. Dots represent actual original observed values. The red solid line shows the median of observed values. The red dashed lines represent the 5th and 95th percentiles of population observed concentrations. The upper and lower purple shaded areas show the 95% confidence intervals for the predicted 5th and 95th percentiles. The middle red shaded area shows the 95% confidence interval for the predicted 50th percentile

GOF diagnostics (Fig. 1) showed that conditional weighted residuals were randomly scattered around zero over time and concentration, indicating no noticeable trends. Observed concentrations closely matched both individual and population predictions. In the pcVPC (Fig. 2), the 5th, 50th, and 95th percentiles of observed concentrations fell within the corresponding 90% prediction intervals from 1,000 simulations, confirming that the model adequately captured both central tendency and variability.

Parameter precision was assessed by non-parametric bootstrap (1,000 data sets resampled at the case level). 897 of the 1,000 bootstrap replicates converged (89.7%), which—given the low information content of these sparse samples—should be interpreted cautiously. Bootstrapped 95% percentile intervals for Ka and CL/F mostly overlapped with the covariance-based 95% confidence intervals (CIs), and the minor deviation at the upper limit confirms that the precision of these parameters is acceptable.

However, for Vd/F, the PI was higher and wider at the upper bound than the CI, which extended much lower. This discrepancy indicates that Vd/F was the least reliably estimated parameter, likely due to sparse early- and late-phase sampling. Overall, the model structure remains supported by GOF and pcVPC plots. Still, the broader—or only partially overlapping—bootstrap intervals suggest limited precision for Vd/F, warranting future validation in larger datasets with richer time-point sampling (Table 2).

### Exposure simulation and dosing evaluation

**Fig. 3:**
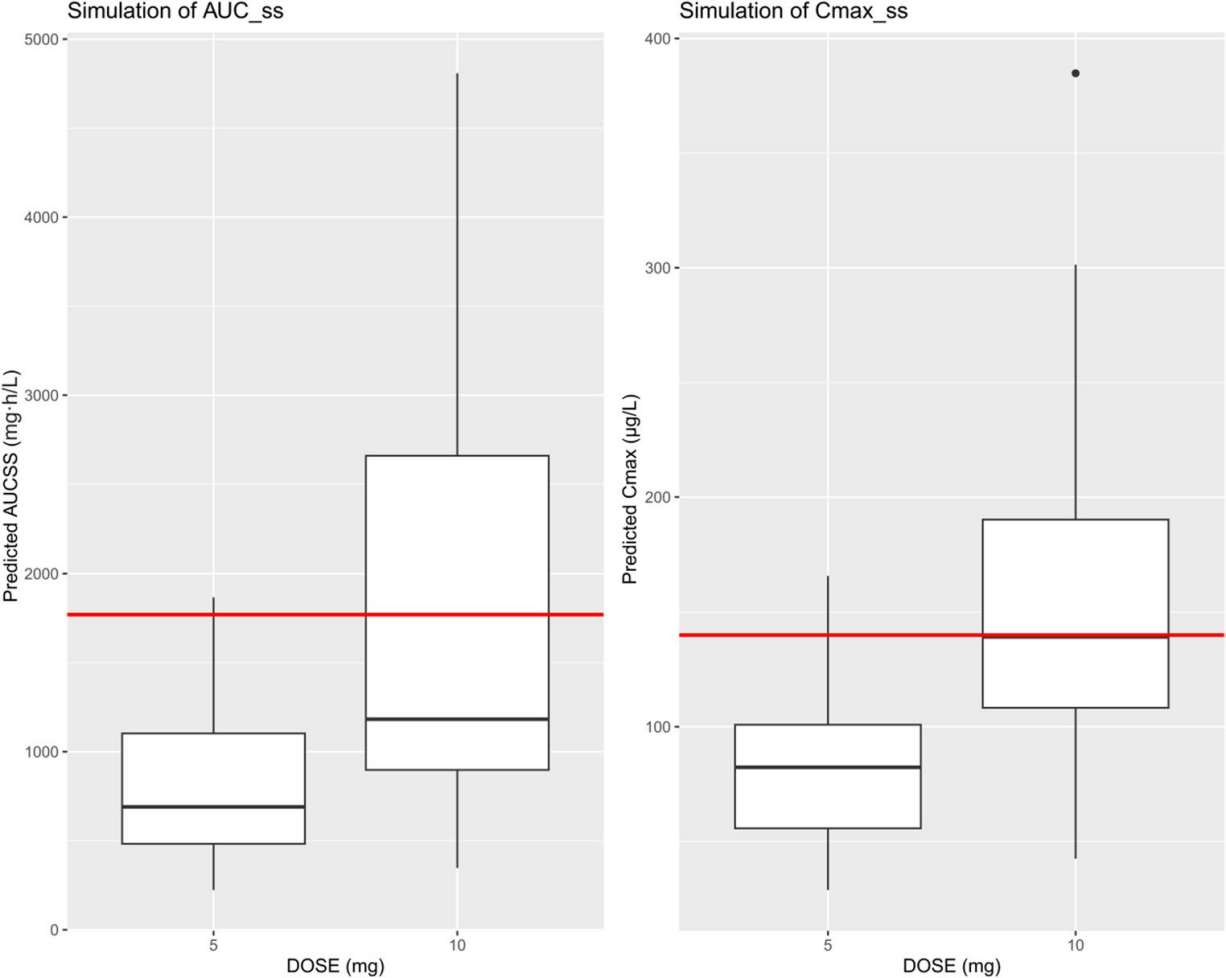
AUC_ss,24_ (left) and C_max, ss_ (right) of 38 patients. The horizontal line within the box represents the median. The lower and upper edges of the box denote the first and third quartiles. The whiskers outside the box represent 1.5 × IQR. Points beyond the whiskers are outliers. The horizontal dashed lines mark the exposure-based safety limits (AUC_ss,24_ = 1.77 mg·h/L; C_max, ss_ = 140 µg/L) [22]

Based on the final PopPK model, patient-specific simulations (*n* = 38) indicated that 96.7% of patients receiving the 5 mg once-daily regimen remained within predefined exposure limits (AUC_ss,24_ ≤ 1.77 mg·h/L; C_max, ss_ ≤ 140 µg/L) [22], versus 62.5% for the 10 mg regimen (Fig. 3).

**Fig. 4:**
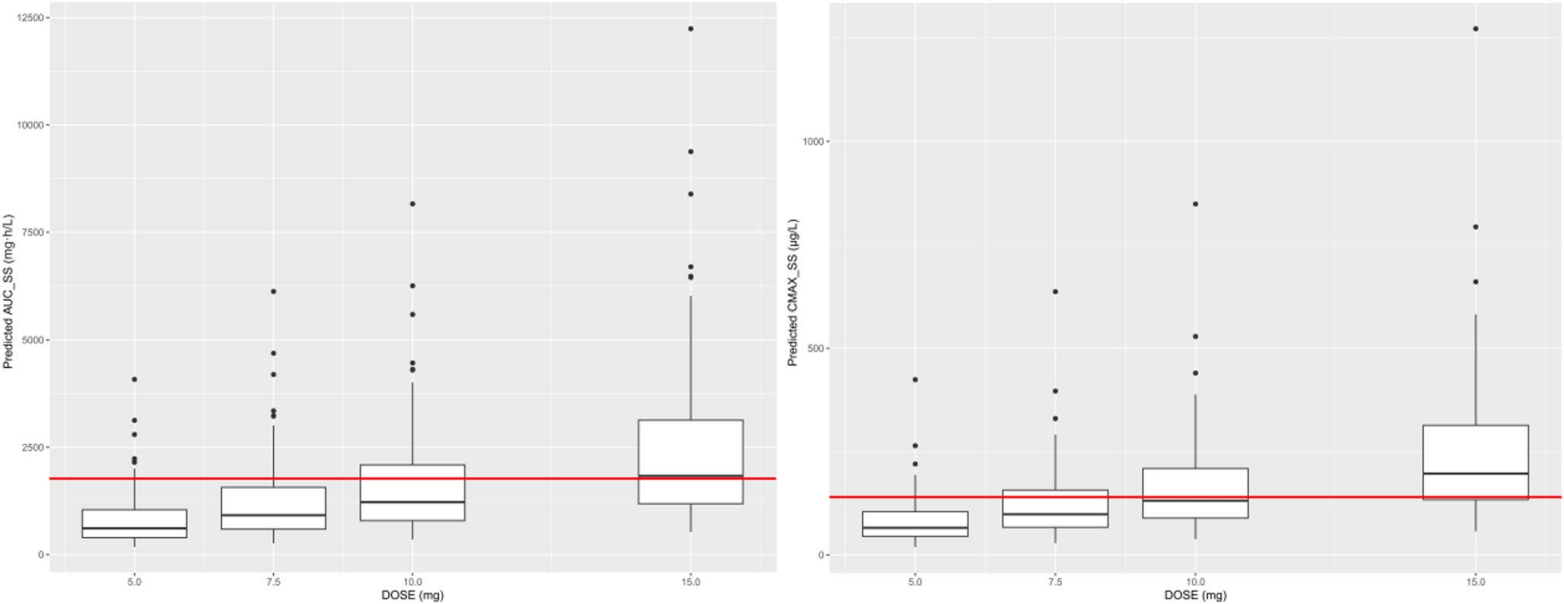
Predicted steady-state exposure distributions for four once-daily rivaroxaban doses in 1,000 virtual post-TIPS patients. Box-and-whisker plots depict (left) total 24-h area under the concentration-time curve at steady state (AUC_ss,24_ ) and (right) steady-state peak plasma concentration (C_max, ss_) for 5, 7.5, 10, and 15 mg regimens. Boxes indicate the interquartile range with the median as a horizontal line; the whiskers outside the box represent 1.5 × IQR, and points beyond are outliers. The horizontal dashed lines mark the exposure-based safety limits (AUC_ss,24_ = 1.77 mg·h/L; C_max, ss_ = 140 µg/L) [22]

In Monte Carlo simulations involving 1,000 virtual post-TIPS patients, the percentages achieving both exposure criteria decreased with increasing dose: 84% at 5 mg/day, 66% at 7.5 mg/day, 55% at 10 mg/day, and 28% at 15 mg/day (Fig. 4).

## Discussion

This is the first study to describe rivaroxaban PK in adults with TIPS and to extrapolate this to exposure-based dosing recommendations. Based on sparsely collected concentration-time data the present analysis provided a one-compartment PopPK model with zero-then first-order absorption, an absorption lag and linear elimination. The model provided satisfactory characterization to 131 plasma observations of the 38 patients, as determined by goodness-of-fit and prediction-corrected visual predictive check. The bootstrap success rate was 89.7%, indicating only moderate model robustness. For most parameters, the bootstrap 95% PI exceeded the 95% CI derived from the covariance step, suggesting that the high variability inherent in the sparse dataset compromised parameter precision and overall model stability. However, pcVPC analysis indicated that the model was reproducing consistently the global exposure distribution that was used for PK prediction. Three key observations could be extracted: (i) The apparent volume of distribution (V_d_/F) 4.75 L was dramatically lower compared to data in non-TIPS patients (21.7–101 L), published by others [18, 23, 24]. Although the 95% bootstrap percentile interval for V_d_/F (1.54–10.3 L) was wider at the upper bound than the covariance-based 95% CI (0.658–9.22 L), it still lay entirely below the non-TIPS range, supporting a true TIPS-specific volume contraction; (ii) There was still considerable unexplained IIV after an extensive covariate search; and (iii) Monte-Carlo simulations suggested that the 5 mg once daily dosing resulted in exposures within safety limits for the vast majority of patients, while dose of 10 mg may warrant therapeutic drug monitoring (TDM) and 15 mg regimen was associated with frequent exceedances of the safety thresholds in simulations [22].

The 78.11 − 95.30% decrease in V_d_/F is biologically plausible in the context of portal-systemic shunting and cirrhosis-related pathophysiology. Diversion of portal flow directly into the systemic circulation may diminish hepatic first-pass extraction [25] and therefore increases oral bioavailability (F) and mathematically depresses Vd/F even if true tissue volume is unchanged. In addition, hepatic blood flow and microsomal enzyme activity (in particular CYP3A4 and CYP2J2) are diminished after TIPS, which may reduce intrinsic clearance and increase circulating concentrations. Together, these mechanisms provide a coherent explanation for the markedly reduced apparent volume of distribution observed in this population. Regarding ascites, we explored baseline ascites status (none/mild/moderate-severe; see Table 1) as a covariate on Vd/F. Ascites was not supported as a significant covariate (*p* > 0.05) and was therefore not retained in the final model, suggesting that, within the range of ascites severity represented in our cohort, ascites alone did not explain the lower Vd/F. This may be biologically plausible because rivaroxaban is highly protein-bound and not primarily distributed into extracellular fluid; ascites may instead act as a proxy for broader decompensation (e.g., hypoalbuminemia, hemodynamic changes) rather than directly increasing distribution volume. Nevertheless, the power to detect an ascites effect was limited by sample size and by under-representation of advanced decompensation, so a modest association cannot be excluded.

Whereas prior PopPK studies in non-shunted populations have consistently observed creatinine clearance (CrCl) as an important predictor of rivaroxaban clearance, no such effect was noted among our post-TIPS subjects [23]. Several factors may account for this discrepancy. First, the CrCl range in the present cohort was relatively narrow (no patient had CrCl < 40 mL/min), limiting statistical power to detect a slope. Second, renal function in cirrhosis and post-TIPS patients may not be accurately reflected by creatinine-based estimates. Serum creatinine levels can be reduced due to sarcopenia and reduced creatinine production, leading to an overestimation of CrCl when calculated using the Cockcroft-Gault formula. The relatively low albumin levels observed in this cohort further support the presence of advanced liver disease, in which creatinine-based renal markers may not accurately reflect renal clearance. Third, portosystemic shunting reduces hepatic blood flow and CYP3A-mediated first-pass extraction, leading to an increase in oral bioavailability (F) and a reduction in the hepatic contribution to total clearance. In this context, even if intrinsic or renal clearance increases, a simultaneous increase in F may offset this effect when clearance is expressed as CL/F, resulting in an apparent lack of change in estimated clearance and a flattened CrCl-exposure relationship. Together, these factors may explain why the renal covariate observed in non-TIPS populations was not reproduced in the present study. Future studies that enroll patients across a wider spectrum of renal function will be needed to delineate the true role of renal clearance after TIPS.

The findings provide valuable reference for anticoagulation therapy in post-TIPS patients. The marked TIPS-specific reduction in apparent volume of distribution (V_d_/F) has important pharmacokinetic implications. With apparent clearance (CL/F) remaining relatively unchanged in the present analysis, For a fixed oral dose, a reduction in apparent volume of distribution (V_d_/F) is associated with a steeper concentration-time profile and higher observed peak concentrations when total concentrations are considered. This elevation in systemic exposure may shift patients toward the higher-risk of major bleeding. Model-based simulations suggested that the 5 mg once-daily regimen maintained drug exposures within safety thresholds for the vast majority of patients, whereas approximately 40% of post-TIPS patients receiving the 10 mg regimen exceeded these limits, which may indicate a clinically meaningful increase in bleeding risk [22]. These results suggest that 5 mg once daily may be a reasonable candidate regimen for further evaluation. The 10 mg regimen should only be considered with intensive therapeutic drug monitoring, and doses exceeding 10 mg/day cannot be recommended based on the current model-based evidence. It should also be noted that the sparse early sampling design limits precise identification of true C_max_. Moreover, rivaroxaban absorption has been described as complex, with multiple concentration peaks consistent with enteroenteric recycling, which further complicates peak estimation [26, 27]. Of particular clinical importance, patients with low rivaroxaban exposure are at increased risk of thromboembolism, while those with high exposure are at risk of experiencing bleeding. This dual risk profile requires clinicians to consider individual patient characteristics (e.g., liver function, concomitant medications) and implement therapeutic drug monitoring for precision dosing in post-TIPS patients.

This study has several limitations. (i) Sample size and sampling density: although 38 patients exceed many single-centre PopPK studies, external validation in larger cohorts is required. Because post-TIPS patients are clinically fragile, a sparse-sampling design (2, 4, 24 h and, in later recruits, 8 h after the observed dose) was used to minimize patient burden; this inevitably reduced the precision of absorption-phase estimates and the power to detect time-dependent model misspecification. (ii) The cohort lacked representation of patients with advanced hepatic dysfunction (CTP class C), limiting our ability to characterize pharmacokinetics in this most severely impaired subgroup. (iii) Sparse covariate diversity: few subjects exhibited severe renal impairment, further limiting detection of covariate effects. (iv) Absence of pharmacodynamic end points: bleeding and thrombotic outcomes were not prospectively recorded, so risk assessment relied on surrogate safety thresholds derived from non-TIPS populations. (v) Lack of anti-Xa calibration: drug exposure was evaluated solely by LC-MS/MS total concentrations; anti-Xa activity—which integrates unbound drug and pharmacodynamic response—was not measured. The exposure thresholds used in this analysis were based solely on exposure–bleeding risk evaluations. Although the U.S. FDA clinical pharmacology review includes exposure–effectiveness analyses, the relationship is weak and lacks a clear inflection point, precluding the establishment of validated AUC- or C_max_-based efficacy targets. Thus, efficacy could not be quantitatively incorporated into dosing assessments. (vi) Genotype omission: polymorphisms in ABCB1, ABCG2 and CYP3A4 that could account for residual variability were not analyzed [19, 28]. (vii) The bootstrap analysis highlighted the 95% CI failed to encompass the corresponding 95% PI for V_d_/F and Ka, emphasising parameter imprecision. (viii) No external validation dataset was available to confirm model performance. These caveats should be borne in mind when interpreting the model and its dosing recommendations; nevertheless, the framework provides a valuable foundation for future precision-dosing research in this vulnerable population.

Future research should: (i) validate the current model in independent post-TIPS cohorts with richer sampling and clinical outcome adjudication; (ii) integrate pharmacogenomics to identify predictors of high exposure; and (iii) develop joint PKPD models incorporating anti-Xa activity, thromboelastography and bleeding events.

## Conclusion

This study establishes the first population pharmacokinetic model specifically for rivaroxaban in post-TIPS patients and confirms a markedly reduced apparent volume of distribution relative to non-TIPS populations. Model-based simulations suggested that the 5 mg once-daily regimen was associated with exposures remaining within predefined safety thresholds in the majority of simulated patients, whereas about 60% of patients receiving 10 mg/day met both the AUC_ss,24_ and C_max, ss_ criteria [22]. This suggests that therapeutic drug monitoring may be concidered when prescribing the 10 mg dose, while doses > 10 mg/day were associated with frequent safety-threshold exceedances in simulations and therefore warrent caution in this population. These findings provide a quantitative, hypothesis-generating basis for individualized rivaroxaban dosing following TIPS from a pharmacokinetic and safety perspective, and may inform future prospective studies and clinical evaluation in this vulnerable patient population.

## Data Availability

De-identified rivaroxaban concentration-time data and NONMEM control streams have been deposited in Zenodo (DOI: 10.5281/zenodo.17035573).
